# miRs-134 and -370 function as tumor suppressors in colorectal cancer by independently suppressing EGFR and PI3K signalling

**DOI:** 10.1038/srep24720

**Published:** 2016-04-20

**Authors:** Sherien M. El-Daly, Mohammed L. Abba, Nitin Patil, Heike Allgayer

**Affiliations:** 1 Department of Experimental Surgery, Medical Faculty Mannheim, University of Heidelberg, Germany; 2 Centre for Biomedicine and Medical Technology (CBTM) Mannheim, University of Heidelberg, Germany; 3 Department of Medical Biochemistry, Medical Division, National Research Centre, Cairo, Egypt

## Abstract

Growth factor receptor signalling plays a central and critical role in colorectal cancer. Most importantly, the EGFR signalling cascade involving PI3K/AKT/mTOR and Raf/MEK/ERK pathways are particularly relevant, since they are commonly activated in several cancer entities, including colorectal cancer. In this study, we show that miRs-134 and -370 are both capable of regulating these pathways by targeting EGFR and PIK3CA. In three different colorectal cancer cell lines (DLD1, HCT-116 and RKO), suppression of EGFR and PIK3CA through the enhanced expression of miR-134 or -370 led to a suppression of the key molecules of the PI3K/AKT/mTOR pathway. Furthermore, overexpression of miR-134 or -370 resulted in a significant reduction of cell proliferation, colony formation, migration, invasion and *in-vivo* tumor growth and metastasis. Concurrent experiments with small interfering RNAs targeting the prime targets show that our selected miRNAs exert a greater functional influence and affect more downstream molecules than is seen with silencing of the individual proteins. Taken together, these data indicate that miRs-134 and -370 are potential tumour suppressor miRNAs and could play a fundamental role in suppressing colorectal cancer tumorigenesis through their ability to co-ordinately regulate EGFR signalling cascade by independently targeting EGFR and PIK3CA.

The burden of colorectal cancer remains significant, as it is still one of the leading causes of cancer-related morbidity and mortality^1^. Distorted signaling events have been shown to contribute immensely to the initiation, progression and dissemination of the disease. In colorectal cancer, EGFR signaling cascades involving PI3K/AKT/mTOR and the Raf/MEK/ERK pathways are not only relevant as they have been implicated in over 50% of tumors, but are also of enormous interest since they have been shown to interact with each other^2^,^3^. The binding of EGF to its receptor and formation of a functionally active EGFR initiates a mitogenic cascade of intracellular signals to the cytoplasm and then to the nucleus. The two major intracellular pathways activated by EGFR are the Ras/Raf mitogen-activated protein kinase (MAPK) pathway, which controls cell proliferation, cell-cycle progression, metastasis and angiogenesis, and the phosphatidylinositol 3 kinase (PI3K)/AKT/mTOR pathway, which activates a wide range of important cellular processes including cell growth, angiogenesis, migration, survival and anti-apoptotic signals^3^,^4^,^5^,^6^. Dysregulated signaling through these pathways is common in colorectal cancer and often it is the result of genetic alterations in key components in these pathways or the upstream activators^7^,^8^,^9^. The overwhelming evidence supporting the critical role of several components of this cascade in cancer has led to concerted efforts to develop inhibitors of some of the key members of this pathway, some of which are in already used in clinical practice^10^.

MicroRNAs are an endogenous group of small non-coding RNAs that regulate protein-coding gene expression by repressing translation or cleaving RNA transcripts. MiRNAs are predicted to regulate up to 30% of all human genes and more than 60% of mammalian protein-coding genes^11^. MiRNAs are involved in all aspects of cell physiology, and have been linked with many human pathological conditions, including cancer^12^. More than 50% of miRNA genes are located in cancer-associated genomic regions or fragile sites, and can act as oncogenes or tumor suppressors depending on their target genes^13^. MicroRNAs are able to target several mRNAs simultaneously, and a single mRNA can be simultaneously targeted by several miRNAs. This property of miRNAs makes them very potent regulators of physiological and pathological processes, and in several instances miRNAs have been implicated as master regulators of critical processes like EMT and metastasis^14^.

In this study, we set out to identify miRNAs that could significantly impact growth factor signaling pathways, particularly PI3K, and since the pathways are invariably interlinked, identify miRNAs that could possibly act on multiple important nodes in the cascade. As a hint, we used a list of miRNAs that we identified to be differentially and significantly expressed in a matched series of resected normal, tumor and metastasis colorectal tissues^15^. A special emphasis was made to identify miRNAs whose expressions were concurrently altered in tumor tissue in relation to normal comparisons, as well as in the metastasis tissue compared to the tumor. In this second focused analysis, a particular subset of miRNAs was identified and fed into in silico tools (TargetScan, miRANDA) to identify predicted targets, which were subsequently grouped into canonical signaling cascades using the Ingenuity Core Analysis pipeline. Amongst other miRs that we already showed to orchestrate a potent EMT/MET-driving network which impacts on metastasis^15^, this algorithm yielded miRs-134 and -370 as the most significant hits, and we were able to show that these two miRNAs directly target EGFR and PIK3CA and in the process modulate both the PI3K/AKT/mTOR and Raf/MEK/ERK pathways with subsequent effects on tumor growth, proliferation, migration and invasion.

## Results

### EGFR and PIK3CA are targeted by miRs-134 and -370 and are upregulated in colorectal cancer cells with low expression of these miRNAs

**Figure 1:**
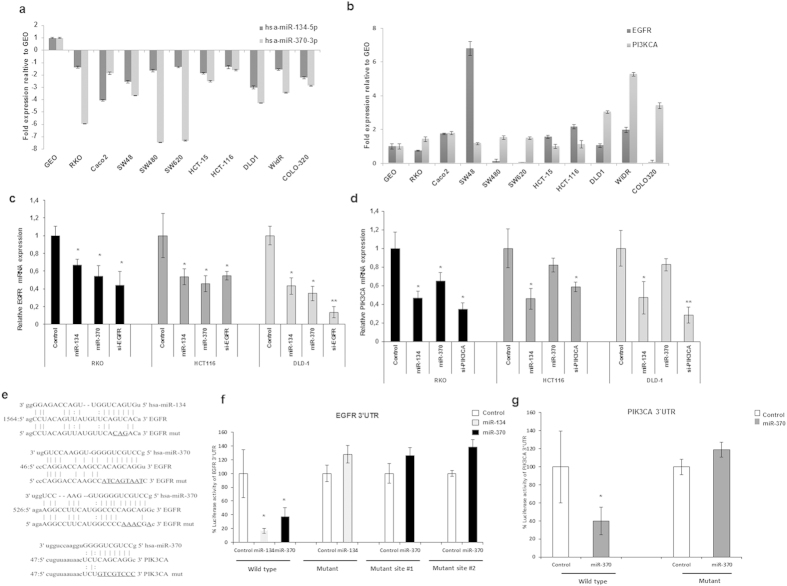
miRs 134 and -370 modulate growth factor signaling by targeting EGFR and PIK3CA. (**a**) Cell line screening for the endogenous expression of miRs-134 and -370 in a panel of colorectal cancer cell lines, graph shows expression relative to the GEO cell line after normalization to RNU6B. (**b**) Expression of EGFR and PIK3CA in the same colorectal cancer cell lines screened for miR-134 and miR-370, expression is shown relative to the GEO cell line. (**c**,**d**) Evaluation of EGFR and PIK3CA mRNA expression following transfection with miR-134, miR-370 or siRNAs against EGFR or PIK3CA (**e**) Alignment of seed sequences of miR-134 and miR-370 with binding motifs in the 3′UTRs of their respective target mRNAs and nucleotides which were mutated (underlined) for luciferase assays; (**f**,**g**), Reporter gene assays showing a direct regulation of EGFR 3′UTR by miR-134 and -370 and PIK3CA by miR-370. Site directed mutagenesis shows elimination of the observed regulation.

To explore novel oncogenic targets of miRs-134 and -370, we used a combination of in silico prediction algorithms (TargetScan, miRANDA and Ingenuity), and consistently EGFR was found to be a highly predicted target for miRs-134 and -370 and PIK3CA to be a highly predicted target for miR-370 (Supplementary Table 1). As a guide to finding out if this prediction was accurate, we screened a panel of colorectal cancer cell lines for the expression of these two miRs as well as the target mRNAs. We found that the expression of these two mRNAs were higher in cell lines with low miRNA expression and vice-versa (p < 0.05, Fig. 1a,b, Supplementary Table 2). Thereafter we proceeded to confirm if the forced expression of these two miRs had any effect on the mRNA of EGFR and PIK3CA using miRNA mimics and siRNAs, as well as appropriate controls, where we discovered that both miR-134 and -370 led to a significant decrease of EGFR and PI3K mRNA in cultured cells (Fig. 1c,d). Based on these results, we sought to demonstrate that these two genes are direct targets of these miRNAs. The 3′UTR of EGFR has two sites for miR-370 and one for miR-134 while PIK3CA harbors one site for miR-370 (Fig. 1e). Reporter gene assays with the wild type and mutated 3′UTR constructs of these mRNAs showed that EGFR is a valid target for miRs-134 and 370 and PIK3CA is a valid targets of miR-370 (Fig. 1f,g, p < 0.05).

### miRs-134 and -370 modulate the PIK3CA/AKT/mTOR and Raf/MEK/ERK pathways

**Figure 2:**
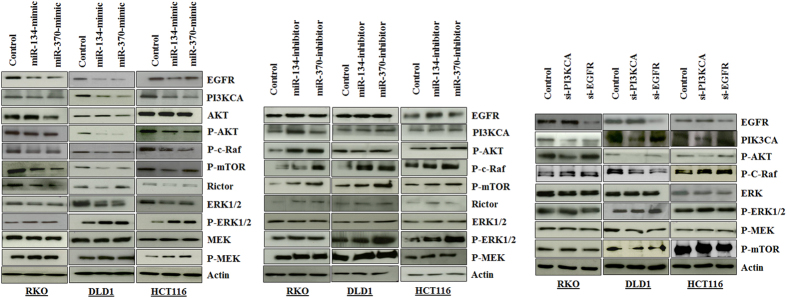
The PIK3CA/AKt/mTOR and Raf/MEK/ERK pathways are regulated by miRs-134 and-370. (**a**) Western blots showing validated targets and key molecules in the PIK3CA/AKT/mTOR and Raf/MEK/ERK signaling cascades following transfection with miR-134 or miR-370. (**a**) mimics; (**b**) Inhibitors, and (**c**) siRNAs in RKO, DLD1 and HCT-116 colorectal cancer cell lines. The probed proteins include EGFR, PIK3CA, AKT, p-Akt, p-c-Raf, p-mTOR, Rictor, ERK 1/2, p-ERK 1/2, MEK and p-MEK. Proteins were evaluated 48–72 hrs after transfection of cell lines, as indicated.

Since EGFR and PIK3CA are both integral components of the PIK3CA/AKT and Raf/MEK/ERK pathways, we assessed the effect of miRs-134 and -370 not only on their individual targets, but also the entire signaling cascade. To evaluate this effect, we independently transfected the mimics and inhibitors of these two miRNAs in three colorectal cancer cell lines (RKO, DLD1 and HCT-116) and assessed protein expression and activitation of the key signaling molecules using Western blots with antibodies directed against whole-peptide and phosphorylated epitopes. Since miRNAs can target several genes simultaneously, we additionally transfected the same cell lines with validated siRNAs specific for the targets, to evaluate if observed changes were mediated, at least in part via EGFR or PIK3CA. For miR-134, we observed a suppression of EGFR and PIK3CA in all three cell lines with a subsequent repression of p-Akt, p-c-Raf, p-mTOR, Rictor and ERK1/2. AKT and MEK were not changed significantly in their protein amounts, however, p-ERK and p-MEK were elevated (Fig. 2a–c). Densitometry results for the Western Blots are provided in Supplementary Table 3). In the case of miR-370, the pattern seen for miR-134 was recapitulated, however not to the same degree as for miR-134. EGFR was not affected by miR-370 in the HCT-116 cell line, whereas significant repression was observed in RKO and DLD1 cell lines. Nonetheless, p-ERK and p-MEK were also elevated in all three cell lines (Fig. 2a–c). With the miRNA inhibitors, the reverse pattern to that of the mimics was observed in the key signaling molecules.

### Enhanced expression of miRs-134 and -370 suppresses cell proliferation and colony formation

**Figure 3:**
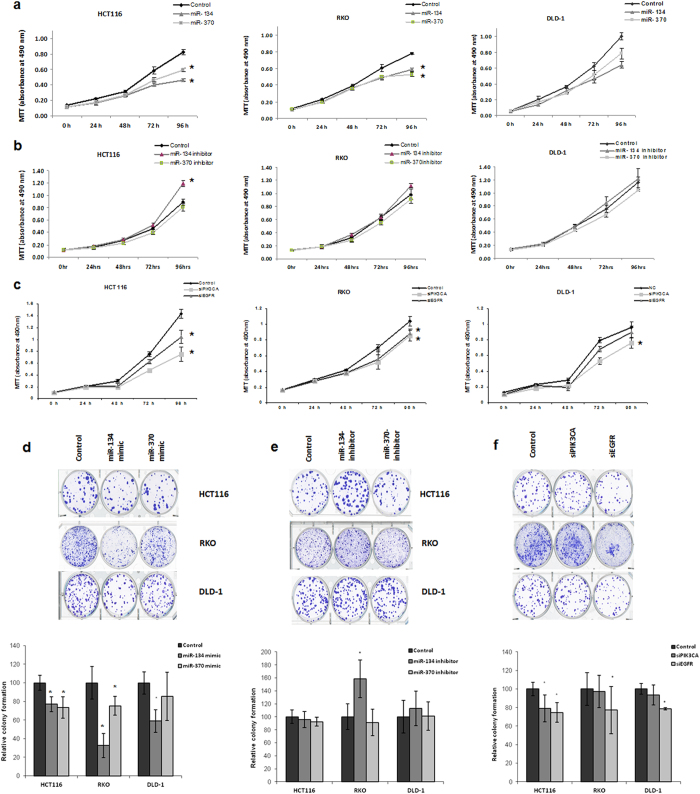
miRs-134 and 370 suppress cell proliferation and colony formation. Cellular growth curves of HCT-116, RKO and DLD1 cell lines following transfection with (**a**) miR-134 or miR-370 mimics; (**b**) miR-134 or miR-370 inhibitors and (**c**) siRNAs targeting EGFR and PIK3CA. A total of 1 × 10^3^ cells/cell line were seeded in 96-well plates and proliferation was assessed at 0, 24, 48, 72 and 96 time points using the MTT assay. Colony formation assay was performed with the same cell lines used for cell proliferation. The cell lines were treated with (**d**) miR-134 or miR-370 mimics; (**e**) miR-134 or miR-370 inhibitors and (**f**) siRNAs targeting EGFR and PIK3CA. The upper panels show representative examples of the scanned plates and the lower panels signify the overall quantification of the colony counts. Details are as discussed in the materials and methods (significance was set at p < 0.05 as compared to control group).

After our observation that miRs-134 and -370 significantly alter the EGFR signaling cascade, we evaluated the functional outcome of this deregulation. Increased proliferation is a major phenotype of activated PI3K/AKT/mTOR signaling, and using mimics and inhibitors of miRs-134 and -370, we conducted an MTT assay over a 96 hr time period in HCT-116, RKO and DLD1 cell lines. With the mimics of miR-134 and -370 and siRNAs against EGFR and PIK3CA, we observed a significant decrease in cell proliferation 96 hrs after transfection as compared to the scrambled transfected controls. With the miRNA inhibitors, the reverse pattern was observed but was significant only for miR-134 in HCT116 (Fig. 3a–c). Similarly, clonogenic assays which evaluate the ability of a single cell to grow and proliferate into a colony were additionally carried out with the same transfection conditions for mimics, inhibitors or siRNAs. Also with this assay, we observed significantly fewer colonies with the transfection of miR-134 and -370 mimics compared to scrambled-sequence controls (Fig. 3d–f). The converse was observed with the miRNA inhibitors. Interestingly, in both proliferation and colonogenic assays, transfection with both EGFR and PIK3CA siRNAs did not yield the same degree of suppression as miR-134 or -370 mimics.

### miRs-134 and -370 suppress *in vitro* migration and invasion

**Figure 4:**
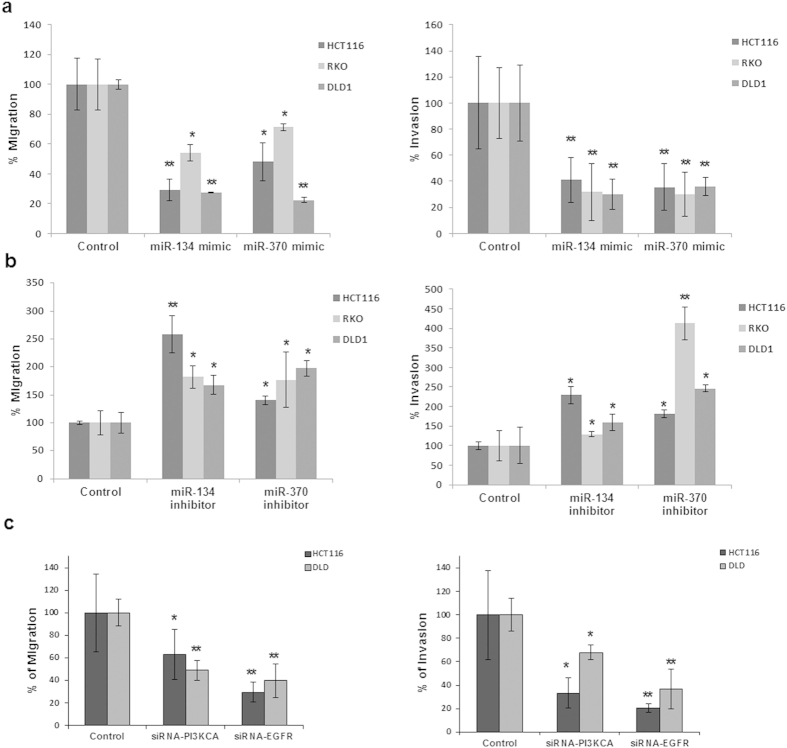
miRs-134 and -370 suppress *in vitro* migration and invasion. (**a**–**c**) Boyden chamber migration and invasion assays in HCT116, RKO and DLD-1 colorectal cancer cell lines treated with miR-134 and miR-370 mimics, miR-134 and miR-370 inhibitors or siRNAs targeting EGFR and PIK3CA, respectively. 5 × 10^4^ and 1 × 10^5^ cells were seeded in transwell chambers for migration and invasion in serum deprived medium and the mobility of cells towards serum containing media was measured by the Cell Titer-Glo assay. Values represent the percentage of migrated cells in relation to the total number of cells.

Although the PI3K pathway is primarily channeled to enhancing survival, proliferation and growth, a number of studies have also shown that this pathway also supports migration, invasion and metastasis via a number of downstream mediators including EIF5A2^16^ and Integrin-linked kinase^17^. Along these lines, we initially investigated the impact of miR-134 and -370 on *in vitro* migration and invasion. Using the Boyden chamber assay, with and without the addition of a matrigel coating, for invasion and migration assays respectively, we were able to show that both miR-134 and miR-370 mimics could significantly suppress migration and invasion in RKO, HCT-116 and DLD-1 colorectal cancer cell lines. Consistently, treatment with the inhibitors of the two miRNAs yielded the opposite effect, where we observed enhanced invasion, especially in DLD-1 cells. Small interfering RNAs (siRNAs) targeting the key signaling molecules; PIK3CA and EGFR also caused a significant decrease in migration and invasion, but interestingly, not to the same extent as with the miRNA mimics (Fig. 4a–c).

### miRs-134 and -370 reduce *in vivo* tumor growth and metastasis in the CAM assay model

**Figure 5:**
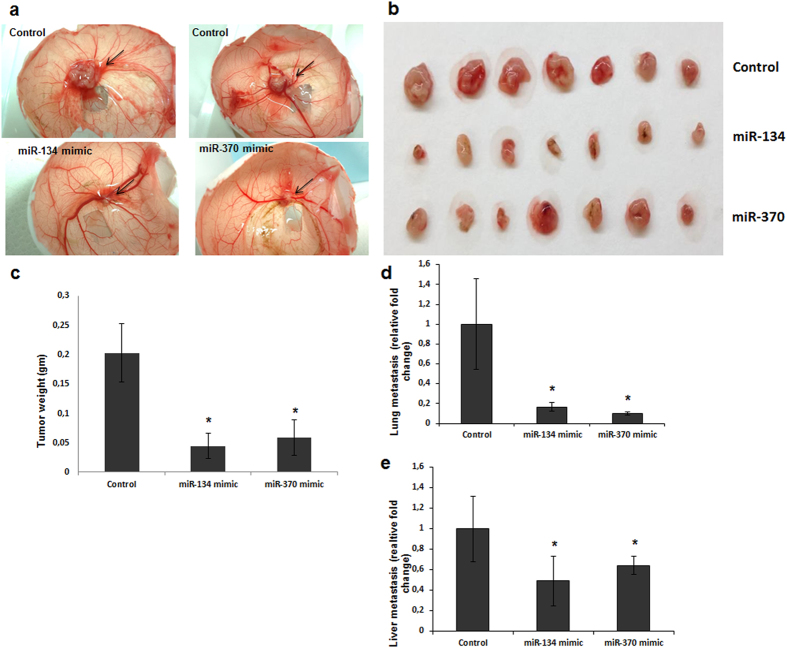
miRs-134 and -370 suppress *in-vivo* tumor growth and metastasis. miR-134, miR-370 (mimic and/or inhibitor) or scrambled -transfected RKO and DLD1 cell lines were inoculated on the upper CAM of 10 day old embryos and following a further 7 days of incubation, tumor growth was evaluated. (**a**) shows in-situ control and miRNA-treated tumors on the upper CAM at the site of inoculation, (**b**) shows the resected tumors from control and miRNA-treated groups arranged alongside each other, n = 7 eggs were used in each arm, (**c**) Average tumor weight in the three groups. (**d**) lung and e) liver metastasis in the CAM model were evaluated quantitatively by RT-PCR using genomic DNA isolated from the harvested organs; p < 0.05 (*) and p < 0.01 (**).

Finally, we sought to assess if these miRs have any effect on tumor growth and distant metastasis *in vivo*. Towards this end, we used our well established CAM assay model^15^,^18^. 7 days after seeding the miRs-transfected cells on the upper chorioallantoic membrane, the chicken embryos were sacrificed, the growing tumors on the upper CAM resected, and the lungs and livers harvested. The resected tumor masses were weighed and evaluated (Fig. 5a,b, Supplementary Fig. 1). Distant metastasis was assessed with human-specific Alu-PCR comparing the miR- with the scrambled control groups (Fig. 5c–e). We observed a significant reduction in the tumor weight with miRNA mimics and enhanced tumor growth with the inhibitors and a 3–4 fold decrease in lung and liver metastasis in the miR-134 and -370 transfected groups compared to the controls (p < 0.05, two tailed Mann-Whitney test, Fig. 5a–e). Enhanced expression and repression of miRNAs was monitored at regular intervals *in-vitro* over the 7 day period (Supplementary Fig. 2). Furthermore, the expression of the key targets, EGFR and PI3KCA in the CAM tumors were also evaluated at the mRNA and protein levels (Supplementary Fig. 3).

### miRs- 134 and -370 are downregulated in resected human tumors and correlates inversely with EGFR and PIK3A expression

**Figure 6:**
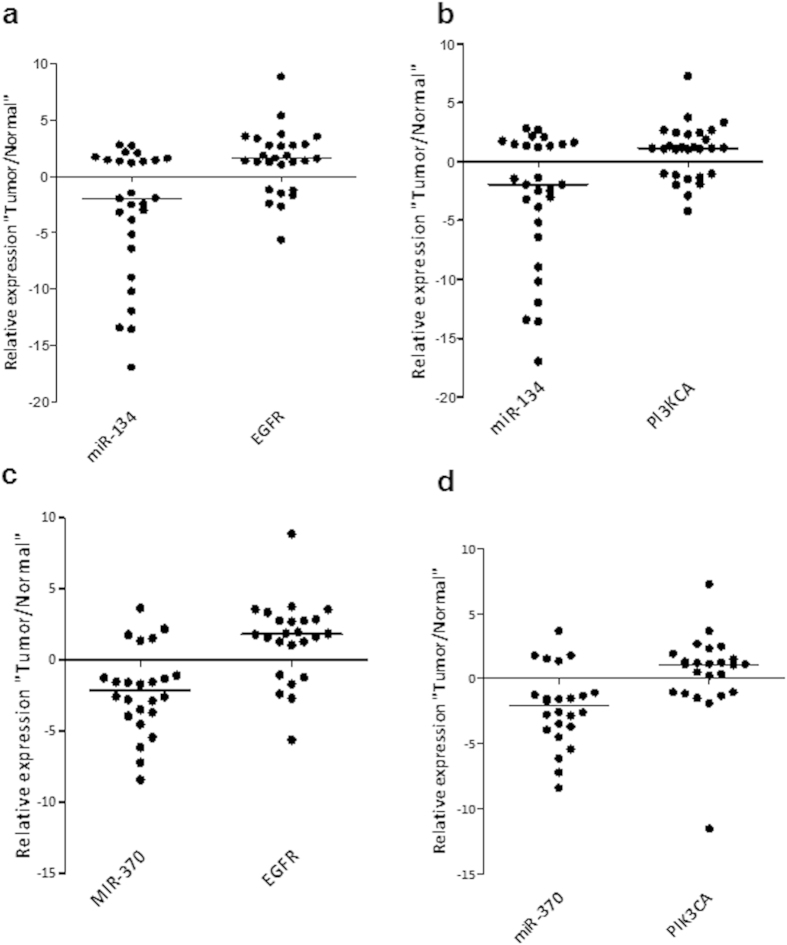
miRs-134 and -370 are downregulated in resected human tumors and correlate inversely with EGFR and PIK3A expression. (**a**–**d)** Dot plots showing the relative tumor/normal expression of miRs-134 and -370 and that of the corresponding mRNA expression of EGFR and PIK3CA in a cohort of 25–30 patients. Both miR-134 and miR-370 are compared with EGFR and PIK3CA. In all cases, a predominant downregulation of the miRNAs together with a corresponding over-expression of the EGFR and PIK3CA mRNAs was observed. Both miRNAs were significantly downregulated in tumor compared to the corresponding normal samples (miR-134, p = 0.0077; miR-370, p = 0.00367).

Following the experimental evidence supporting the significant impact of miRs-134 and 370 in modulating several tumorigenic processes, including metastasis, we decided to analyze the *in vivo* significance of these two miRNAs in human resected tumors. We screened a matched tumor/normal tissue series of (n = 30) resected colorectal cancer tissues for the expression of miRs-134 and -370 and observed a significant downregulation in tumor, compared to the corresponding normal samples. Likewise, we screened the prime targets of these miRNAs (EGFR and PIK3CA) and discovered that they were both significantly upregulated in the tumor tissues (Fig. 6a–d).

## Discussion

MicroRNAs play a crucial role in the regulation of many tumor- and metastasis- associated processes. The fundamental challenge has been, and still is, the identification and validation of the key regulators of distinct (pathological) biological processes within relevant contexts^15^,^19^,^20^. Using a genome-wide miRNA array screen of resected normal, tumor and corresponding metastasis tissues, we found miRs-134 and -370 to be significantly downregulated in tumor and metastasis tissues. These two miRNAs have been reported to be closely located on chromosome 14q32 in the DLK1-DIO3 imprinting genomic region which harbors one of the largest miRNA clusters in the genome and has been identified as a cancer-associated genomic region^21^.The differential regulation of one or both of miRs-134 and -370 has been reported in different cancer types including endometrial, lung, glioblastoma and osteosarcoma, as well as in hepatocellular carcinoma, gastrointestinal stromal tumors, laryngeal squamous cell carcinoma, leukemia and prostate cancer^22^,^23^,^24^,^25^,^26^,^27^,^28^,^29^,^30^,^31^,^32^ but to the best of our knowledge, not in colorectal cancer.

In the present study, we identified and validated EGFR and PIK3CA as novel targets of miRs-134 and-370 in CRC. We observed a significant reduction in luciferase reporter activity of both 3′UTRs when we treated cell lines with the corresponding miRNA-mimics, the effect of which was abrogated when the seed-sequence binding motif was mutated. This was paralleled by a reduction in the mRNA and protein expression of EGFR and PIK3CA.

EGFR is a cell surface tyrosine kinase transmembrane receptor and a member of the ErbB family. The binding of EGF to its receptor enables EGFR to form homo- and heterodimers that initiate autophosphorylation of the intracellular domain through tyrosine kinase activity subsequently activating several downstream signaling pathways, including the PI3K/AKT/mTOR, Ras/Raf/MAPK, JNK, and the Stat3/Stat5 pathways^2^,^33^. These pathways converge to enhance DNA synthesis, cell proliferation, angiogenesis, invasion, and metastasis. Deregulation in the EGFR pathway contributes to the progression of several solid tumors including CRC and clinically, up to 80% of CRC patients have been reported to have overexpression of EGFR that is commonly associated with poor response rates, advanced tumor stages and increased risk of metastasis^34^,^35^. Likewise, PI3K also activated by growth factor stimulation through receptor tyrosine kinases RTKs (including EGFR) and G-protein coupled receptors (GPCRs) is also associated with malignant behavior including cell growth, migration, and survival^36^. Several studies have reported a cross-talk between the PI3K/Akt/mTOR and Ras/Raf/MEK/ERK pathways. Both are activated through the same upstream receptor proteins like EGFR, HER2 and, PDGFR^3^,^37^,^38^ and a number of studies have reported negative feedback loops in this cross-talk^39^,^40^.

In this study, we show that EGFR and PIK3CA are effectively targeted by miRs-134 and -370 with a consequential effect on downstream signaling molecules. Western blots following transfection with mimics showed not only a reduction in the expression of the primary targets EGFR and PIK3CA, but also the key molecules of the PI3K/AKT/mTOR and Raf/MEK/ERK pathways including p-AKT, p-mTOR, Rictor, p-c-Raf and ERK1/2. Surprisingly we observed enhanced phosphorylation of MEK1/2 and ERK1/2 proteins. This paradoxical effect has been reported before and was attributed to a negative feedback loop whose components differ slightly based on context. In HER2 overexpressing cell lines, the inhibition of PI3K induces the dimerization of HER2/3 and increases the binding of the GRB2 to HER2 and the p85 subunit of PI3K to HER3 subsequently inducing ERK phosphorylation^41^. A similar compensatory feedback response was also observed using the mTOR inhibitor (RAD001). Inhibition of mTOR1 was found to induce MAPK activation through a feedback mechanism dependent on S6K1/PI3K/RAS, where the inhibitory effect of S6K1 on PI3K is unleashed when mTORC1 is inhibited resulting in an increased signal toward the RAS/MEK/ERK cascade^42^. Accordingly, the additional use of MEK inhibitors was found to suppress this compensatory feedback activation, since MEK is the immediate upstream activator of ERK^41^,^42^,^43^,^44^.

Consequently, we were able to demonstrate that forced overexpression of miRs-134 and -370 significantly suppressed cell proliferation, colony formation and migration/invasion in 3 different CRC cell lines. This observed anti-tumor effect was more pronounced with the miRNAs than with targeted silencing of the EGFR and PIK3CA proteins using validated siRNAs. These findings suggest that congruent to their mechanism of action, these miRNAs, to some extent, still target other proteins in the EGFR signaling cascade, and as such end up with a more potent silencing of the pathway than with either EGFR or PI3KCA individually. A similar outcome has been reported for miR-134 in glioblastoma by Zhang and colleagues where they also observed significantly reduced proliferation and survival^24^. Moreover, we also provided *in vivo* evidence for the tumor suppressor effects of miRs-134 and -370 using the CAM chicken embryo model, which has been validated in several studies as a valuable model for monitoring tumor growth, invasion and metastasis^15^,^18^,^45^. The results of this assay reveal the promising effect of miRs-134 and-370 in not only reducing tumor growth, but in also suppressing distant metastasis of tumor cells. A very interesting observation from our results is that despite the activation of ERK, which is tumorigenic, functionally, we were still able to demonstrate a significant decrease in tumorigenic properties of the cancer cells in terms of proliferation, colony formation, invasion and even metastasis. While this supports a potential advantage that miR-134 and miR-370 as novel tumor suppressors in CRC have over single-molecule targeting agents, it in no way underscores the relevance that the additional targeting of MEK will have in further suppressing tumor growth and dissemination.

## Materials and Methods

### Cell lines and culture

The RKO, CaCo2, SW48, SW480, SW620, HCT15, HCT116, WidR, DLD-1, Colo-320 (human colorectal), and 239T (mouse embryonic kidney) cell lines were obtained from the American Type Culture Collection (ATCC). The GEO cell line was a kind gift from Douglas Boyd (MD Anderson Cancer Center). The cell lines were maintained in the recommended media supplemented with 10% FBS and 1% penicillin/streptomycin. All cells were cultured in a humidified incubator at 37 °C with 5% CO2.

### RNA extraction, cDNA synthesis and RT-PCR

Total RNA including miRNA was extracted and purified from cell lines and snap-frozen colorectal cancer tissues using Qiagen’s miRNeasy Mini Kit (Hilden, Germany) according to the manufacturer’s instructions. Colorectal cancer tissues were obtained from the tumor bank of the Mannheim Medical Faculty, University of Heidelberg, Germany and their use was approved by the Ethical Committee of the Medical Faculty, University Hospital Mannheim.

RNA was reverse transcribed using the M-MuLV Reverse Transcription Kit (Fermentas) and the miScript reverse transcription Kit (Qiagen) for mRNA and miRNA respectively. Real-time PCR was performed using Quantitect SYBR Green PCR reagents on a LightCycler 480 (Roche). All samples were normalized to the internal control (β -actin or RNU6) and fold changes were calculated with the 2^-ΔΔCt^ method. All experiments were performed in accordance with approved guidelines and regulations.

RT-PCR primers were purchased from Qiagen Quantitect collection; PI3KCA (Cat# QT00014861), EGFR (Cat# QT00085701), β-actin (Cat# QT00095431) and the primers for miRNAs were also purchased from Qiagen, miR-134_2 (Cat# MS00031437), miR-370_1 (Cat# MS0004053) and for internal control RNU6-2_11 (Cat# MS00033740).

### 3′UTR cloning, site-directed mutagenesis and reporter gene assays (EGFR and PIK3CA)

The 3′UTR of PIK3CA was PCR amplified with the forward 5′-TACTCTAGACCACACAATTAAACAGCATGCA-3′ and reverse 5′-TACCCTAGGGGCATAACATGAAATTGCGCA-3′ primer pair, while that of EGFR was amplified with forward 5′-TACTCTAGAACGGAGGATAGTATGAGCCC-3′ and reverse 5′-TACCCTAGGAATGCTGTAGGGGCTCTGAC-3′ primer combination. Both amplicons were independently cloned into the pLightSwitch 3′UTR vector (Switch Gear Genomics, USA) between the XbaI/XmaJI (AvrII) restriction sites. The 3′UTR of PIK3CA harbors one site for miR-370 while EGFR has two sites for miR-370 and one for miR-134. Mutant constructs for the single miR-370 site in PIK3CA, the two sites in EGFR and the single site for miR-134 were generated with the site-directed mutagenesis kit (Agilent Technologies) using the wild type constructs as template (primer sequences are given in Supplementary Table 4).

For luciferase reporter assays, 293T cells were transiently transfected with wild type or mutant reporter plasmid in the presence of miR-370, miR-134 or scrambled control oligonucleotide together with a Renilla luciferase control vector (for normalization). Luciferase activity was determined 24 hrs after transfection with the Dual-Luciferase Reporter Assay System (Promega, Madison, WI, USA).

### Transient transfection using miRNA mimics, inhibitors or siRNAs

Three different colorectal cancer cell lines (HCT116, RKO and DLD-1) were used for transfection with miRNA-mimics, -inhibitors, siRNAs or their corresponding scrambled controls. MiR-134 and miR-370 mimics and inhibitors were purchased from Ambion, Life Technologies (miR-134-5p mimic ID: MC10341 and inhibitor ID: MH10341) and (miR-370-3p mimic ID: MC12868 and inhibitor, ID MH12868). Validated siRNAs targeting EGFR (Cat. # AM16708) and PIK3CA (Cat. # AM51331) were also obtained from Ambion.

Mimics and siRNAs were transfected at a final concentration of 120 nM and miRNA inhibitors at a final concentration of 150 nM using the Lipofectamine TM 2000 transfection reagent (Invitrogen). The cells were incubated for 16–18 hrs following transfection before proceeding with experiments.

### Cell proliferation assay

Cell proliferation was determined with the MTT [3-(4,5-dimethylthiazol-2-yl)-2,5-diphenyl- 2H-tetrazolium bromide] assay. Briefly HCT116, RKO and DLD-1 colorectal cancer cell lines transfected with miRNA mimics, inhibitors, siRNAs of PIK3CA/EGFR or corresponding scrambled controls were seeded in 96-well plates at a density of 3×10^3^ cells/well in a total volume of 100 μl of medium with 10% (v/v) FBS. Eight replicates were made for each condition and each evaluated time point. 20 ul of MTT reagent (0.5 mg/ml, Sigma, St. Louis, MO) was added to each well and the resulting formazan crystals solubilized in 100 ul of 10%SDS/0.01 M HCL. The absorbance was measured using a microplate reader (TECAN Trading AG, Switzerland) at 570 nm with a reference wavelength of 650 nm. Cell proliferation was evaluated over a period of 96 hrs.

### Colony formation assay

HCT116, RKO and DLD-1 cells were transected with mimics, inhibitors, siRNAs or their corresponding scrambled controls. 16–18 hrs after transfection, the cells were trypsinized and re-seeded at a density of 400–600 cells/well in a 6 well plate and maintained in their corresponding media containing 10% FBS at 37 °C. After 14 days, the colonies were fixed with methanol and stained with 0.1% crystal violet for 15 min, and the ensuing colonies scanned and counted.

### *In vitro* migration and invasion Assay

Migration and invasion assays were performed using transwell Boyden chambers (8.0-μm pore size, Corning Incorporated, USA) without or with a matrigel coating, respectively. Following transfection with mimics, inhibitors or siRNAs, 5 × 10^4^ and 1 × 10^5^ cells were used for migration and invasion assays, respectively. Cells were re-suspended in serum-free medium and placed in the transwell. Complete medium with 10% fetal bovine serum was added to the lower chamber as a chemo-attractant. After 24-hour incubation at 37 °C in 5% CO_2_, both migrated/invaded cells on the lower surface of the membrane and the non-migrated/invaded cells in the upper chamber were detached with trypsin, transferred to 96 well plates (Nunc), and the luminescent signals measured with CellTiterGlo on a microplate reader (TECAN Trading AG, Switzerland). For each well, relative migration/invasion was calculated by dividing the migrated/invaded cell values with the combined total for the well.

### Western blotting

Total cell protein lysates were prepared with RIPA lysis buffer with proteinase and phosphatase inhibitors. Protein concentration was determined with the BCA kit (Pierce, Rockford, IL, USA). For each sample, 25 ug of protein were separated on a 10% SDS–PAGE gel and transferred to a nitrocellulose membrane. After blocking, the blots were incubated with primary antibodies raised against; EGFR, PIK3CA, p-Akt (Ser473), Akt, p-c-Raf (Ser259), p-mTOR (Ser2448), Rictor (53A2), p-p44/42 MAPK “p-ERK1/2” (Thr202/Tyr204), p-MEK1/2 (Ser217/221) all purchased from Cell Signaling Technology and β-actin rabbit mAb (Abcam, Cambridge, UK). The blots were subsequently incubated with the corresponding horseradish peroxidase-conjugated secondary antibodies and detected by chemi-luminescence (Millipore, Billerico, Massachusetts, USA).

### CAM assay

The CAM assay was conducted as previously reported (15,18). Briefly, 2 × 10^6^ RKO/DLD1 cells transiently transfected with either miR-134 or miR-370 mimics, inhibitors or scrambled control oligonucleotides were seeded on the upper chorioallantoic membrane of 10-day-old chicken embryos after artificially creating a miniaturized window on the shell. The window was then covered with cellotape and the eggs incubated without rotation for a further 7 days in the incubator. On the 17th day of the experiment, the egg shells were cut open along the latitudinal axis in the midline with a pair of dissecting scissors, and the chicken embryos decapitated. The lungs and liver were harvested and placed immediately into 15 ml tubes containing tissue lysis buffer. The developing tumors on the upper CAM were carefully dissected out of the CAM membrane and weighed. Genomic DNA was isolated from the liver and lungs and the number of metastasized cells evaluated using human specific Alu-PCR PCR as detailed in the PCR section^18^.

### Statistical analysis

The differences between groups were statistically analysed using two tailed unpaired and paired t–tests when dealing with independent (cell lines, before/after treatment) and dependent (tumor/normal patient) variables respectively. All experiments were carried out at least in triplicate and at least in three independent attempts. Calculations were made using Sigmaplot, Microsoft Excel and Graphpad prism 5.0 tools. Data were considered to be statistically significant when p < 0.05 and represented graphically as p < 0.05 (*) and p < 0.01 (**).

## Additional Information

**How to cite this article**: El-Daly, S. M. *et al*. miRs-134 and -370 function as tumor suppressors in colorectal cancer by independently suppressing EGFR and PI3K signalling. *Sci. Rep*. **6**, 24720; doi: 10.1038/srep24720 (2016).

## Supplementary Material

Supplementary Information

Supplementary Dataset 1

## References

[b1] Siegel R. L., Miller K. D. & Jemal A. Cancer statistics, 2015. CA: a cancer journal for clinicians 65, 5–29, doi: 10.3322/caac.21254 (2015). 25559415

[b2] Scaltriti M. & Baselga J. The epidermal growth factor receptor pathway: a model for targeted therapy. Clinical cancer research : an official journal of the American Association for Cancer Research 12, 5268–5272, doi: 10.1158/1078-0432.CCR-05-1554 (2006). 17000658

[b3] Steelman L. S. . Roles of the Raf/MEK/ERK and PI3K/PTEN/Akt/mTOR pathways in controlling growth and sensitivity to therapy-implications for cancer and aging. Aging 3, 192–222 (2011). 21422497 10.18632/aging.100296 PMC3091517

[b4] Kolch W. & Pitt A. Functional proteomics to dissect tyrosine kinase signalling pathways in cancer. Nature reviews. Cancer 10, 618–629, doi: 10.1038/nrc2900 (2010). 20720570

[b5] Zhao L. & Vogt P. K. Class I PI3K in oncogenic cellular transformation. Oncogene 27, 5486–5496, doi: 10.1038/onc.2008.244 (2008). 18794883 PMC2757120

[b6] Meloche S. & Pouyssegur J. The ERK1/2 mitogen-activated protein kinase pathway as a master regulator of the G1- to S-phase transition. Oncogene 26, 3227–3239, doi: 10.1038/sj.onc.1210414 (2007). 17496918

[b7] Neumann J. . Alterations in the EGFR pathway coincide in colorectal cancer and impact on prognosis. Virchows Archiv : an international journal of pathology 463, 509–523, doi: 10.1007/s00428-013-1450-0 (2013). 23934607

[b8] De Roock W. . Effects of KRAS, BRAF, NRAS, and PIK3CA mutations on the efficacy of cetuximab plus chemotherapy in chemotherapy-refractory metastatic colorectal cancer: a retrospective consortium analysis. The Lancet. Oncology 11, 753–762, doi: 10.1016/S1470-2045(10)70130-3 (2010). 20619739

[b9] Wang J. . Colon carcinoma cells harboring PIK3CA mutations display resistance to growth factor deprivation induced apoptosis. Molecular cancer therapeutics 6, 1143–1150, doi: 10.1158/1535-7163.MCT-06-0555 (2007). 17363507

[b10] Spano J. P. . Epidermal growth factor receptor signaling in colorectal cancer: preclinical data and therapeutic perspectives. Annals of oncology : official journal of the European Society for Medical Oncology/ESMO 16, 189–194, doi: 10.1093/annonc/mdi057 (2005). 15668269

[b11] Lee Y., Jeon K., Lee J. T., Kim S. & Kim V. N. MicroRNA maturation: stepwise processing and subcellular localization. The EMBO journal 21, 4663–4670 (2002). 12198168 10.1093/emboj/cdf476 PMC126204

[b12] Utikal J., Abba M., Novak D., Moniuszko M. & Allgayer H. Function and significance of MicroRNAs in benign and malignant human stem cells. Seminars in cancer biology, doi: 10.1016/j.semcancer.2015.07.001 (2015). 26192966

[b13] Calin G. A. . Human microRNA genes are frequently located at fragile sites and genomic regions involved in cancers. Proceedings of the National Academy of Sciences of the United States of America 101, 2999–3004, doi: 10.1073/pnas.0307323101 (2004). 14973191 PMC365734

[b14] Farazi T. A., Spitzer J. I., Morozov P. & Tuschl T. miRNAs in human cancer. The Journal of pathology 223, 102–115, doi: 10.1002/path.2806 (2011). 21125669 PMC3069496

[b15] Mudduluru G. . A Systematic Approach to Defining the microRNA Landscape in Metastasis. Cancer research 75, 3010–3019, doi: 10.1158/0008-5472.CAN-15-0997 (2015). 26069251

[b16] Khosravi S. . Role of EIF5A2, a downstream target of Akt, in promoting melanoma cell invasion. British journal of cancer 110, 399–408, doi: 10.1038/bjc.2013.688 (2014). 24178756 PMC3899752

[b17] Delcommenne M. . Phosphoinositide-3-OH kinase-dependent regulation of glycogen synthase kinase 3 and protein kinase B/AKT by the integrin-linked kinase. Proceedings of the National Academy of Sciences of the United States of America 95, 11211–11216 (1998). 9736715 10.1073/pnas.95.19.11211 PMC21621

[b18] van der Horst E. H., Leupold J. H., Schubbert R., Ullrich A. & Allgayer H. TaqMan-based quantification of invaive cellss in the chick embryo metastasis assay. BioTechniques 37, 940–942, 944, 946 (2004). 15597543 10.2144/04376ST02

[b19] Osaki M., Okada F. & Ochiya T. miRNA therapy targeting cancer stem cells: a new paradigm for cancer treatment and prevention of tumor recurrence. Therapeutic delivery 6, 323–337, doi: 10.4155/tde.14.122 (2015). 25853308

[b20] Ye J. J. & Cao J. MicroRNAs in colorectal cancer as markers and targets: Recent advances. World journal of gastroenterology 20, 4288–4299, doi: 10.3748/wjg.v20.i15.4288 (2014). 24764666 PMC3989964

[b21] Benetatos L. . The microRNAs within the DLK1-DIO3 genomic region: involvement in disease pathogenesis. Cellular and molecular life sciences : CMLS 70, 795–814, doi: 10.1007/s00018-012-1080-8 (2013). 22825660 PMC11114045

[b22] Gao Y., Liu T. & Huang Y. MicroRNA-134 suppresses endometrial cancer stem cells by targeting POGLUT1 and Notch pathway proteins. FEBS letters 589, 207–214, doi: 10.1016/j.febslet.2014.12.002 (2015). 25528443

[b23] Kitamura K. . MiR-134/487b/655 cluster regulates TGF-beta-induced epithelial-mesenchymal transition and drug resistance to gefitinib by targeting MAGI2 in lung adenocarcinoma cells. Molecular cancer therapeutics 13, 444–453, doi: 10.1158/1535-7163.MCT-13-0448 (2014). 24258346

[b24] Zhang Y. . Multiple receptor tyrosine kinases converge on microRNA-134 to control KRAS, STAT5B, and glioblastoma. Cell death and differentiation 21, 720–734, doi: 10.1038/cdd.2013.196 (2014). 24440911 PMC3978301

[b25] Sarver A. L. . MicroRNAs at the human 14q32 locus have prognostic significance in osteosarcoma. Orphanet journal of rare diseases 8, 7, doi: 10.1186/1750-1172-8-7 (2013). 23311495 PMC3566973

[b26] Yin C. . Hepatocyte nuclear factor-4alpha reverses malignancy of hepatocellular carcinoma through regulating miR-134 in the DLK1-DIO3 region. Hepatology 58, 1964–1976, doi: 10.1002/hep.26573 (2013). 23775631

[b27] Haller F. . Localization- and mutation-dependent microRNA (miRNA) expression signatures in gastrointestinal stromal tumours (GISTs), with a cluster of co-expressed miRNAs located at 14q32.31. The Journal of pathology 220, 71–86, doi: 10.1002/path.2610 (2010). 19768731

[b28] Li J. . miR-134 inhibits epithelial to mesenchymal transition by targeting FOXM1 in non-small cell lung cancer cells. FEBS letters 586, 3761–3765, doi: 10.1016/j.febslet.2012.09.016 (2012). 23010597

[b29] Chen X. P., Chen Y. G., Lan J. Y. & Shen Z. J. MicroRNA-370 suppresses proliferation and promotes endometrioid ovarian cancer chemosensitivity to cDDP by negatively regulating ENG. Cancer letters 353, 201–210, doi: 10.1016/j.canlet.2014.07.026 (2014). 25063739

[b30] Yungang W., Xiaoyu L., Pang T., Wenming L. & Pan X. miR-370 targeted FoxM1 functions as a tumor suppressor in laryngeal squamous cell carcinoma (LSCC). Biomedicine & pharmacotherapy = Biomedecine & pharmacotherapie 68, 149–154, doi: 10.1016/j.biopha.2013.08.008 (2014). 24055400

[b31] Xu W. P. . Perturbation of MicroRNA-370/Lin-28 homolog A/nuclear factor kappa B regulatory circuit contributes to the development of hepatocellular carcinoma. Hepatology 58, 1977–1991, doi: 10.1002/hep.26541 (2013). 23728999

[b32] Zhang X. . The tumor suppressive role of miRNA-370 by targeting FoxM1 in acute myeloid leukemia. Molecular cancer 11, 56, doi: 10.1186/1476-4598-11-56 (2012). 22900969 PMC3533721

[b33] Oda K., Matsuoka Y., Funahashi A. & Kitano H. A comprehensive pathway map of epidermal growth factor receptor signaling. Molecular systems biology 1, 2005 0010, doi: 10.1038/msb4100014 (2005). 16729045 PMC1681468

[b34] Spano J. P. . Impact of EGFR expression on colorectal cancer patient prognosis and survival. Annals of oncology : official journal of the European Society for Medical Oncology/ESMO 16, 102–108, doi: 10.1093/annonc/mdi006 (2005). 15598946

[b35] Porebska I., Harlozinska A. & Bojarowski T. Expression of the tyrosine kinase activity growth factor receptors (EGFR, ERB B2, ERB B3) in colorectal adenocarcinomas and adenomas. Tumour biology : the journal of the International Society for Oncodevelopmental Biology and Medicine 21, 105–115, doi: 30116 (2000). 10686540 10.1159/000030116

[b36] Yuan T. L. & Cantley L. C. PI3K pathway alterations in cancer: variations on a theme. Oncogene 27, 5497–5510, doi: 10.1038/onc.2008.245 (2008). 18794884 PMC3398461

[b37] Shimizu T. . The clinical effect of the dual-targeting strategy involving PI3K/AKT/mTOR and RAS/MEK/ERK pathways in patients with advanced cancer. Clinical cancer research : an official journal of the American Association for Cancer Research 18, 2316–2325, doi: 10.1158/1078-0432.CCR-11-2381 (2012). 22261800

[b38] Mendoza M. C., Er E. E. & Blenis J. The Ras-ERK and PI3K-mTOR pathways: cross-talk and compensation. Trends in biochemical sciences 36, 320–328, doi: 10.1016/j.tibs.2011.03.006 (2011). 21531565 PMC3112285

[b39] McCubrey J. A. . Ras/Raf/MEK/ERK and PI3K/PTEN/Akt/mTOR cascade inhibitors: how mutations can result in therapy resistance and how to overcome resistance. Oncotarget 3, 1068–1111 (2012). 23085539 10.18632/oncotarget.659 PMC3717945

[b40] McCubrey J. A. . Therapeutic resistance resulting from mutations in Raf/MEK/ERK and PI3K/PTEN/Akt/mTOR signaling pathways. Journal of cellular physiology 226, 2762–2781, doi: 10.1002/jcp.22647 (2011). 21302297

[b41] Serra V. . PI3K inhibition results in enhanced HER signaling and acquired ERK dependency in HER2-overexpressing breast cancer. Oncogene 30, 2547–2557, doi: 10.1038/onc.2010.626 (2011). 21278786 PMC3107390

[b42] Carracedo A. . Inhibition of mTORC1 leads to MAPK pathway activation through a PI3K-dependent feedback loop in human cancer. The Journal of clinical investigation 118, 3065–3074, doi: 10.1172/JCI34739 (2008). 18725988 PMC2518073

[b43] Migliardi G. . Inhibition of MEK and PI3K/mTOR suppresses tumor growth but does not cause tumor regression in patient-derived xenografts of RAS-mutant colorectal carcinomas. Clinical cancer research : an official journal of the American Association for Cancer Research 18, 2515–2525, doi: 10.1158/1078-0432.CCR-11-2683 (2012). 22392911

[b44] Britten C. D. PI3K and MEK inhibitor combinations: examining the evidence in selected tumor types. Cancer chemotherapy and pharmacology 71, 1395–1409, doi: 10.1007/s00280-013-2121-1 (2013). 23443307

[b45] Deryugina E. I. & Quigley J. P. Chick embryo chorioallantoic membrane model systems to study and visualize human tumor cell metastasis. Histochemistry and cell biology 130, 1119–1130, doi: 10.1007/s00418-008-0536-2 (2008). 19005674 PMC2699943

