# Supplementary material for: miRs-134 and -370 function as tumor suppressors in colorectal cancer by independently suppressing EGFR and PI3K signalling
Source: Sci Rep. 2016 Apr 20;6:24720. doi: 10.1038/srep24720 (PMC4837379; doi:10.1038/srep24720)
Supplement: Supplementary Information [file srep24720-s1.doc]

Supplementary Information

miRs-134 and -370 function as tumor suppressors in colorectal cancer by independently suppressing EGFR and PI3K signalling

Sherien M. El-Daly, Mohammed L. Abba, Nitin Patil and Heike Allgayer


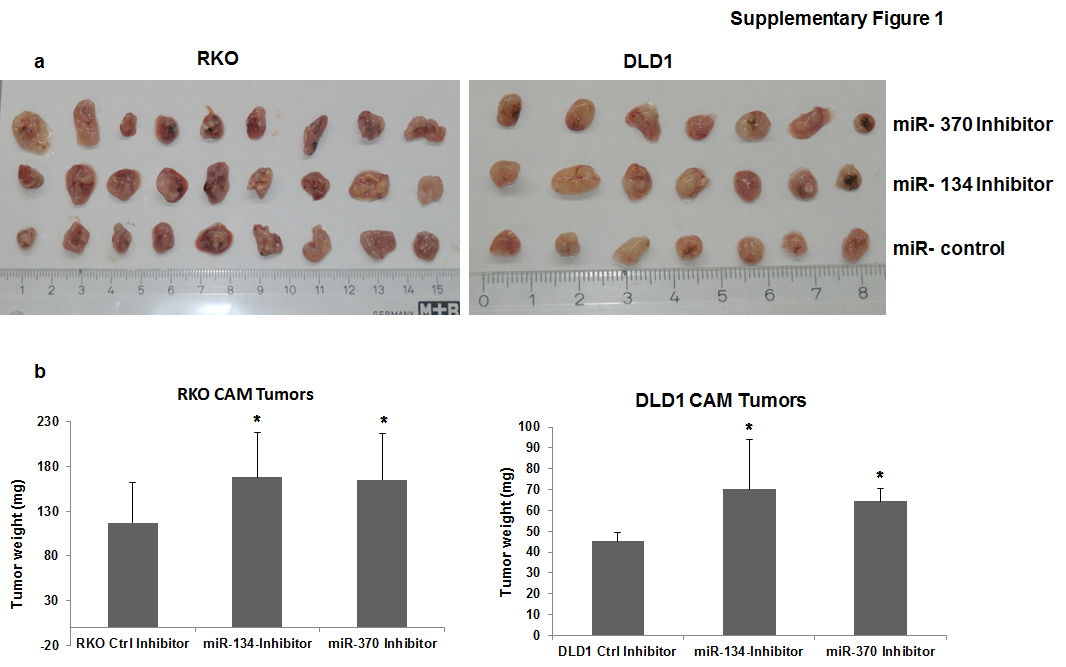


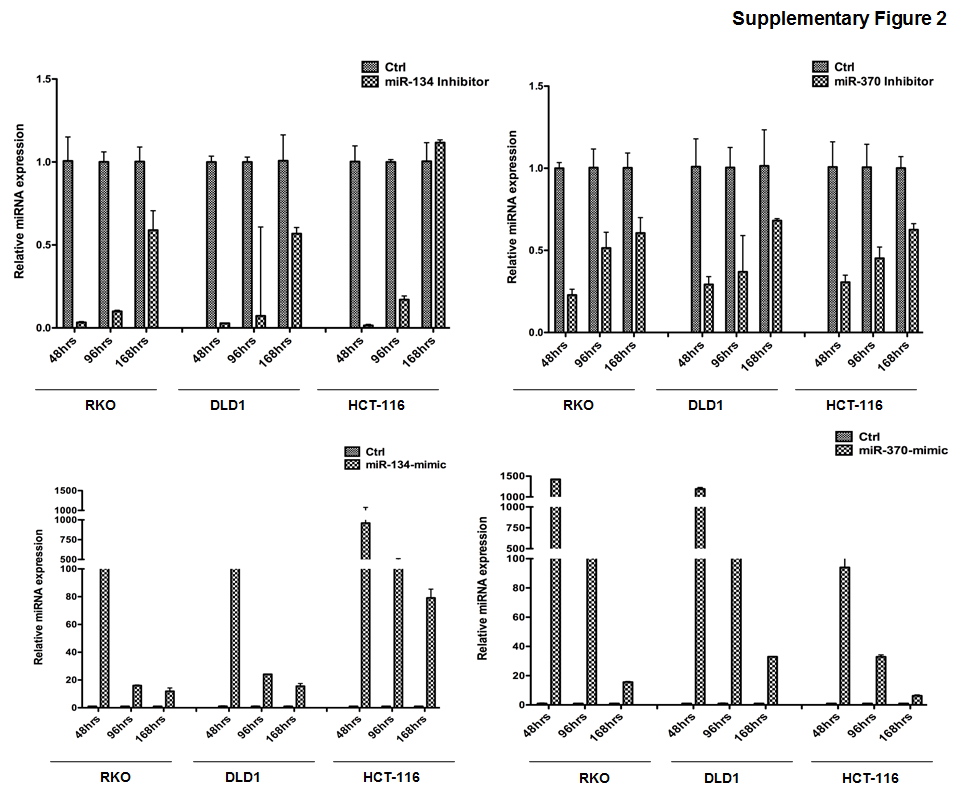


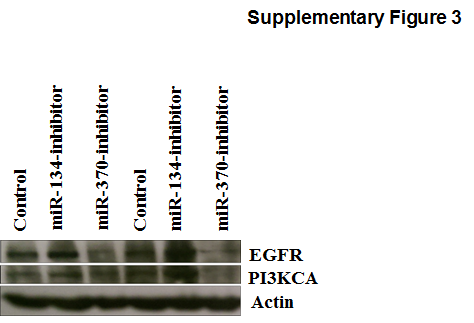


Supplementary Figure Legends

**Supplementary Figure 1**: miR-134 and miR-370 inhibitor or scrambled -transfected RKO and DLD1 cell lines were inoculated on the upper CAM as described in the materials and methods. Figure 1a shows the excised tumors in from the control, miR-134 and miR-370 groups in the two cell lines and Figure 1b shows the average weight of these tumors in mg; p < 0.05 ( ⃰).

**Supplementary Figure 2**: Evaluation of miR mimic and Inhibitor expression. The expression levels of both miR-134 and miR-370 (mimic and inhibitor) transiently transfected RKO, DLD1 and HCT-116 cell lines were evaluated intermittently at 48hrs, 96hrs and 168hrs (7 days) after transfection. Cells were seeded in 12 well plates, culture media was changed every 48hrs and the cells were harvested at the designated time points . RNA was isolated from the cell pellets collected at these time points followed by reverse transcription and real time PCR as described in the materials and methods. RT-PCR data was assessed with the delta-delta –CT method using scrambled transfected cells as controls and Sno-72 for internal normalization.

**Supplementary Figure 3**: EGFR and PI3KCA expression in CAM tumors. DLD1 and RKO CAM resected tumors treated with miR-134 and miR-370 inhibitors and were shredded using Qiagen Tissue disruptor. Subsequently protein lysates were made using the described protocol. Western blots show enhanced expression of EGFR and PI3KCA in both cell line tumors with miR-134 inhibitors.

**Supplementary Table 2**: Ct values for endogenous expression of miR-134 and miR-370 in colorectal cancer cell lines

| Cell line | miR-370 | miR-134 |
| --- | --- | --- |
| RKO | 21,64 | 23,9333333 |
| Caco2 | 19,3333333 | 24,8766667 |
| WidR | 20,9966667 | 24,2733333 |
| SW480 | 21,9433333 | 24,1733333 |
| HCT116 | 20,52 | 24,0266667 |
| COLO-320 | 20,6366667 | 24,6733333 |
| SW48 | 20,945 | 24,8366667 |
| HCT15 | 20,655 | 24,65 |
| Geo | 20,25 | 24,6666667 |
| SW620 | 22,15 | 24,1333333 |
| LOVO | 20,6066667 | 25,13 |
| DLD-1 | 21,28 | 25,185 |

**Supplementary Table 3**: Normalized densitometry figures for WB expression in Figure 2 of manuscript. The result for each protein was normalized to the expression of the control which is denominated as 1. The expression of mimic/inhibitor/siRNA transfectants was calculated relative to this value. All calculations were cell line and protein specific.

|  |  | **RKO** |  |  | **DLD1** |  |  | **HCT-116** |  |
| --- | --- | --- | --- | --- | --- | --- | --- | --- | --- |
|  | **Control** | **miR-134 mimic** | **miR-370 mimic** | **Control** | **miR-134 mimic** | **miR-370 mimic** | **Control** | **miR-134 mimic** | **miR-370 mimic** |
| **EGFR** | 1 | 0,46 | 0,52 | 1 | 0,3 | 0,38 | 1 | 0,6 | 0,7 |
| **PI3KCA** | 1 | 0,8 | 0,8 | 1 | 0,4 | 0,32 | 1 | 0,6 | 0,54 |
| **AKT** | 1 | 1,2 | 0,62 | 1 | 1,1 | 0,8 | 1 | 1,2 | 1 |
| **p-AKT** | 1 | 0,8 | 0,8 | 1 | 0,2 | 0,2 | 1 | 0,4 | 0,4 |
| **p-craf** | 1 | 0,63 | 0,6 | 1 | 0,5 | 0,8 | 1 | 0,3 | 0,2 |
| **p-mTOR** | 1 | 0,58 | 0,46 | 1 | 0,38 | 0,69 | 1 | 0,5 | 0,8 |
| **Rictor** | 1 | 0,68 | 0,3 | 1 | 0,4 | 1,2 | 1 | 0,5 | 0,6 |
| **Erk1/2** | 1 | 0,8 | 0,8 | 1 | 0,2 | 0,2 | 1 | 0,54 | 0,61 |
| **pErk1/2** | 1 | 1,4 | 1,28 | 1 | 4 | 4,2 | 1 | 6,3 | 6,1 |
| **p-MEK** | 1 | 2,1 | 1,8 | 1 | 2,3 | 2 | 1 | 2,1 | 2,7 |
|  |  |  |  |  |  |  |  |  |  |
|  |  |  |  |  |  |  |  |  |  |
|  |  | **RKO** |  |  | **DLD1** |  |  | **HCT-116** |  |
|  | **Control** | **miR-134 Inhibitor** | **miR-370 Inhibitor** | **Control** | **miR-134 Inhibitor** | **miR-370 Inhibitor** | **Control** | **miR-134 Inhibitor** | **miR-370 Inhibitor** |
| **EGFR** | 1 | 3 | 1,8 | 1 | 1,9 | 2,1 | 1 | 3 | 1,58 |
| **PI3KCA** | 1 | 3,8 | 2 | 1 | 1,52 | 1,63 | 1 | 1,2 | 1,23 |
| **p-AKT** | 1 | 5,8 | 5,6 | 1 | 0,9 | 1,1 | 1 | 1,5 | 1,5 |
| **p-craf** | 1 | 1,3 | 4,7 | 1 | 7,7 | 6,9 | 1 | 1,8 | 2,5 |
| **p-mTOR** | 1 | 2,7 | 3,6 | 1 | 2,3 | 4,1 | 1 | 1,3 | 2,4 |
| **Rictor** | 1 | 1,5 | 1,46 | 1 | 1,1 | 1,2 | 1 | 1,5 | 0,9 |
| **Erk1/2** | 1 | 1,1 | 1 | 1 | 0,98 | 1,12 | 1 | 1,2 | 1,2 |
| **pErk1/2** | 1 | 3,1 | 2,4 | 1 | 1,6 | 2,8 | 1 | 2,3 | 3,5 |
| **p-MEK** | 1 | 1,52 | 1,48 | 1 | 1,9 | 1,8 | 1 | 1,4 | 1,5 |
|  |  |  |  |  |  |  |  |  |  |
|  |  |  |  |  |  |  |  |  |  |
|  |  | **RKO** |  |  | **DLD1** |  |  | **HCT-116** |  |
|  | **Control siRNA** | **si-PI3KCA** | **si-EGFR** | **Control siRNA** | **si-PI3KCA** | **si-EGFR** | **Control siRNA** | **si-PI3KCA** | **si-EGFR** |
| **EGFR** | 1 | 1,1 | 0,2 | 1 | 1,04 | 0,1 | 1 | 1 | 0,5 |
| **PI3KCA** | 1 | 0,5 | 0,85 | 1 | 0,3 | 0,8 | 1 | 0,6 | 0,9 |
| **p-AKT** | 1 | 0,52 | 1,1 | 1 | 0,08 | 0,9 | 1 | 0,38 | 1,1 |
| **p-craf** | 1 | 1,2 | 1,1 | 1 | 0,7 | 0,58 | 1 | 1,7 | 2,3 |
| **Erk1/2** | 1 | 0,97 | 1 | 1 | 1 | 1,1 | 1 | 0,9 | 0,7 |
| **pErk1/2** | 1 | 1,5 | 1,3 | 1 | 1,2 | 1,4 | 1 | 1,7 | 1,4 |
| **p-MEK** | 1 | 1 | 1,1 | 1 | 0,8 | 0,82 | 1 | 1, 2 | 1,2 |
| **p-mTOR** | 1 | 1,4 | 1 | 1 | 0,8 | 0,9 | 1 | 1,5 | 0,9 |

**Supplementary Table 4**: Sequences of the mutation primers used in the study

| PI3KCA Forward primer | 5'-gtgcaattcctatgcaatcggtctttgggacgacagagttattaacagtgcagtgtggaatccag-3' |
| --- | --- |
| PI3KCA Reverse primer | 5'-ctggattccacactgcactgttaataactctgtcgtcccaaagaccgattgcataggaattgcac-3' |
| EGFR Forward primer “for miR-134” | 5'-acattttgtatgtgtgtgtctgtgaacataactgtaggctgaaaatattttctgaac-3' |
| EGFR Reverse primer“for miR-134” | 5'-gttcagaaaatattttcagcctacagttatgttcacagacacacacatacaaaatgt-3' |
| EGFR Forward primer “1^st^ binding site of miR-370 | 5'-ggctgttgggatggaggattactgatggcttggtcctgggtatcgaaagagtctgg-3' |
| EGFR Reverse primer “1^st^ binding site of miR-370 | 5'-ccagactctttcgatacccaggaccaagccatcagtaatcctccatcccaacagcc-3' |
| EGFR Forward primer “2^nd^ binding site of miR-370 | 5'-gatccggtcgtttggggccatgaaggccttctt-3' |
| EGFR Reverse primer “2^nd^ binding site of miR-370 | 5'-aagaaggccttcatggccccaaacgaccggatc-3' |
